# Empathy and event related potentials before and after EEG based neurofeedback training in autistic adolescents

**DOI:** 10.1038/s41598-025-16767-y

**Published:** 2025-08-22

**Authors:** Julia Fietz, Gabriel Auer, Paul Plener, Luise Poustka, Lilian Konicar

**Affiliations:** 1 Max Planck Institute of Psychiatry, Munich, Germany; 2 International Max Planck Research School for Translational Psychiatry, Munich, Germany; 3 Department of Child and Adolescent Psychiatry, Medical University of Vienna, Währingergürtel 18-20, 1090 Vienna, Austria; 4 Comprehensive Center for Pediatrics (CCP), Medical University of Vienna, Vienna, Austria; 5 Comprehensive Center for Clinical Neuroscience and Mental Health (C3NMH), Medical University of Vienna, 1090 Vienna, Austria; 6 Department of Child and Adolescent Psychiatry and Psychotherapy, Ulm University, Ulm, Germany; 7 Department of Child and Adolescent Psychiatry and Psychotherapy, University Medical Center Göttingen, Göttingen, Germany

**Keywords:** Autism spectrum disorder, Children and adolescents, Neurofeedback, Event-related potentials (ERP), empathy, emotion regulation, Autism spectrum disorders, Emotion, Empathy

## Abstract

Autism Spectrum Disorder (ASD) is often characterized by deficits in emotion regulation and empathic abilities, potentially linked to alterations in prefrontal brain regions. This randomized, controlled clinical trial examines the efficacy of slow cortical potential neurofeedback training, specifically targeting these prefrontal areas, in improving emotion regulation and empathy among children and adolescents with ASD. The study involved 41 participants, with 21 undergoing slow cortical potential training and 20 receiving treatment as usual. All participants were allowed to continue usual care in progress, if it was kept stable. Emotional processing was evaluated using an adapted and extended version of the Multifaceted Empathy Test, alongside electroencephalography assessments focusing on event-related potentials, including N170, LPP, and P300 components. The main findings indicate a significant group × time interaction in P300 latency, with shorter latencies in the SCP neurofeedback group and longer latencies in controls, though post hoc tests were not significant. A trend toward reduced P300 amplitude in the experimental group suggests possible modulation of attentional processing. Additionally, changes in a late component of LPP amplitude were linked to reaction time in processing positive emotions, with increases associated with slower responses and decreases with faster responses. These results suggest slow cortical potential neurofeedback training may influence cognitive efficiency and emotional processing in autistic individuals. While promising, further research is needed to confirm these findings and optimize neurofeedback protocols for this population.

**Supplementary Information:**

The online version contains supplementary material available at 10.1038/s41598-025-16767-y.

## Introduction

Empathy is an emotional response to another’s observed emotional state with an awareness that the source of the experienced emotion is separate from one’s own. Empathy encompasses both affective and cognitive components. Affective empathy involves sharing another’s emotions, while cognitive empathy refers to understanding them^1^.

The ability to manage one’s own emotions, both deliberately and automatically, is known as emotion regulation^2^. According to Thompson et al.^3^, empathy and emotion regulation interact dynamically, influencing how we perceive and respond to others’ emotions. Thompson and colleagues propose that emotion regulation mechanisms can affect empathic processes differently, meaning they may either enhance or suppress empathy. Both empathy and emotion regulation are recognized as crucial for emotional well-being and social functioning, and both have been found to be impaired in autistic individuals^4–6^.

*Empathic competencies*, such as understanding, sharing and responding to others’ emotions, are repeatedly shown to be disturbed in Autism Spectrum Disorder (ASD)^7–11^. Those with higher ASD scores exhibit greater difficulties in recognizing and understanding their own emotions (i.e., alexithymia), which further hinders them in understanding the emotions of others. While these cognitive empathy deficits in autism are well documented, studies have suggested that affective empathy may not differ significantly between autistic individuals and controls^7–10^. However, a recent meta-analysis encompassing a broader range of studies has found significant deficits in both components of empathy in ASD compared to controls, with a large effect size for cognitive empathy and a moderate effect size for affective empathy^11^.

Emotion regulation difficulties, specifically in monitoring and modulating emotions (i.e., emotional dysregulation), are a core characteristic of ASD, alongside deficits in social communication and interaction^12,13^. Autistic children and adolescents often rely more on maladaptive emotion regulation strategies than their typically developing (TD) peers^14^, which contributes to heightened levels of anxiety, depression, and negative emotions in general. These difficulties also interfere with empathic competencies^15^, supporting the view of Thompson et al.^3^ that emotion regulation mechanisms can affect empathic processes. Thus, empathy and emotion regulation are both impaired in ASD, and their dynamic interplay may underlie and/or exacerbate these impairments.

Furthermore, deficits in emotion regulation and empathic competencies are not only evident in early childhood but tend to intensify with age, adding complexity to the emotional and social challenges faced by autistic individuals^16,17^, and underscoring the need for effective interventions. A focus on underlying mechanisms may offer a more consistent framework for understanding and addressing this disorder.

Neuroimaging studies have identified overlapping neural activation patterns for both empathic and emotion regulation processes in subcortical regions (e.g., amygdala, ventral striatum, and insula^18–22^ and cortical regions (e.g., prefrontal cortex, temporoparietal junction, superior temporal sulcus, anterior temporal pole^23–25^ and the anterior cingulate cortex (ACC)^26,27^ ). This overlap suggests that the interactions between empathy and emotion regulation, as described by Thompson et al.^3^, may also be reflected at the neural level.

Notably, atypical activation of subcortical regions such as the amygdala and the nucleus accumbens has been observed in ASD during emotion regulation processes^28^, while insula dysfunction has been linked to impaired empathizing in autistic individuals^29^. Additionally, within cortical regions, the dorsolateral prefrontal cortex has been implicated in deficits related to emotion regulation^28^. Similarly, atypical activation of the temporoparietal junction^30^ and the temporal pole^31^ has been reported during mentalizing, a key component of cognitive empathy. Furthermore, anatomic-functional abnormalities in the sulcus temporalis superior have been associated with impairments in social cognition processes, which underlie empathy among other functions^32^. Finally, reduced activity in the ACC has been observed in ASD during the processing of emotional faces, a fundamental precursor to empathy^33^.

Taken together, these findings suggest that while empathy and emotion regulation share overlapping neural mechanisms, ASD is characterized by atypical activation patterns and structural abnormalities within these regions, which likely contributes to deficits in both domains. Importantly, the affected cortical and subcortical regions are key components of the broader social brain network, a network of brain regions involved in social cognition and behavior. Dysfunction within this network has been consistently associated with social impairments in ASD^34^, including difficulties in empathy and emotion regulation, and is thought to underlie core characteristics of the condition.

While Magnetic Resonance Imaging studies offer valuable insights into the spatial aspects of brain activity in ASD, electroencephalography (EEG) is more accessible, provides significantly higher temporal resolution, and directly measures neural activity, making it a crucial tool for assessing neural function. Evidence from event-related potentials (ERP), which have been linked to both empathic and emotion regulatory processes, further supports the notion of shared neural mechanisms underlying these functions. The neural underpinnings of components such as the N170, the P300 and the Late Positive Potential (LPP) involve as well a complex network of cortical and subcortical regions. These include the amygdala, precuneus, cingulate cortex, insula but also supplementary motor cortex, superior temporal sulcus, frontal gyrus, temporoparietal junction and the prefrontal cortex^35–37^. Importantly, these regions are also key components of the social brain network, as stated above, and exhibit atypical functioning in autistic individuals.

In detail, the N170 is associated with facial emotion recognition, with the strongest amplitudes elicited by anger, fear and happiness^38^, and shows a diminished or absent response to non-facial stimuli^39^. Notably, while the N170’s negative peak typically occurs approximately 170 ms after stimulus onset in adults, it is often delayed in children and adolescents, appearing between 150 and 300 ms. This likely reflects a developmental trend, characterized by a non-linear decrease in amplitude and latency in response to facial stimuli during early childhood^40–42^. Furthermore, higher empathy, as measured by the Interpersonal Reactivity Index^43^was related to a more pronounced (i.e., more negative) N170 amplitude in the right posterior temporal region, particularly in response to happy, angry, surprised, and fearful faces^44,45^. In autistic individuals, numerous studies have reported diminished N170 amplitudes and prolonged latencies compared to TD individuals^42,46–53^. McPartland and colleagues^51^ reported that autistic individuals who exhibited better face recognition abilities had longer N170 latencies, whereas TD individuals with better face recognition showed shorter latencies. The authors concluded that autistic individuals process faces differently, requiring more time for accurate facial recognition, which may explain their slower face processing. A recent meta-analysis overlapped in their findings regarding delayed N170 latencies in response to faces; however, N170 amplitudes were similar between individuals with and without ASD, particularly when attentional demands were involved^54,55^. Similar to findings for autistic adults, studies in autistic children also report delayed N170 latencies during face processing compared to TD children^42^. While attempts to link these results with symptom severity did not reach statistical significance^42^, they were associated with lower IQ and poorer face memory^56^. These findings suggest that N170 responses in individuals with ASD are marked by delayed latencies and, in some cases, reduced amplitudes, reflecting atypical face processing. This may contribute to their difficulties in facial recognition, a well-documented deficit in ASD^57^, which is likely linked to deficits in social interactions^58^. Given that higher empathy has been associated with more pronounced N170 amplitudes^44,45^, it seems conceivable that the reduced amplitudes observed in some individuals with ASD might be associated to the repeatedly shown deficits in empathic competencies^7–11^.

The most robust electrophysiological correlates of empathy—particularly in the context of pain—have been reported for the P300 and LPP^59^. The P300, a positive peak occurring approximately 300 ms post-stimulus, is not only involved in emotion recognition and processing^60,61^, but also plays a role in emotion regulation. Evidence suggests that individuals who habitually employ the adaptive emotion regulation strategy of reappraisal exhibit stronger P300 reactivity during the regulation phase^62^. In autistic individuals, a meta-analysis revealed a reduced P300 amplitude, suggesting potential deficits in decision-making processes^63^, although no differences in P300 latency were observed between ASD and TD groups^63^. Additionally, autistic adolescents and young adults have shown reduced P300 peak latencies, which have been linked to deficits in social awareness, sensation avoidance, and attentional characteristics^64^. Moreover, recent studies also support the findings of reduced P300 amplitude in autistic children without developmental delay^65^. In contrast, Keifer and colleagues^66^ reported heightened P300 amplitudes associated with greater ASD symptomatology, while Magnuson and colleagues^67^ did not find any differences between autistic children and TD children in task performance, P300 amplitude or latency. These findings indicate that P300 responses in ASD are inconsistent, with some studies reporting reduced amplitudes and others showing elevated amplitudes linked to symptom severity. Despite these differences, the overall atypicality of P300 in ASD may contribute to affective impairments, given its role in emotion recognition, processing, and regulation.

Functionally and temporally related to the P300, the LPP is associated with higher-order attentional processes toward affective stimuli^60,61^ as well as to emotion regulation strategies^68–71^. Starting about 400 ms after the stimulus, greater LPP amplitudes to emotionally salient compared to neutral stimuli were already reported in children from the age of 6 years^72^. In terms of empathic competencies, higher self-reported empathy scores have been linked to more pronounced early LPPs (i.e. more positive amplitudes between 300 and 600 ms) in parietal regions, as well as increased late LPPs (i.e. more positive amplitudes between 600 and 800 ms) in frontal regions^44,45^.

Findings regarding the role of LPP in emotion regulation are mixed, with variations depending on the specific emotion regulation strategy and the timing component (early vs. late LPP). Studies have reported both increased and decreased LPP amplitudes^71,73,74^. In autistic children, relatively smaller LPP amplitudes in response to social stimuli (i.e. faces) and larger LPP amplitudes to nonsocial stimuli (common interests such as electronics, vehicles, etc.)^75^ have been observed. Moreover, individuals with high autism scores exhibited stronger LPP responses to human touch compared to control images, with regression models revealing that larger LPP amplitudes during social touch observation accurately predicted higher autistic trait levels^76^. Consistent with these findings, Keifer and colleagues reported that a greater ASD symptomatology was associated with enlarged LPPs^66^. These findings suggest that LPP responses in ASD are atypical and may contribute to difficulties in empathy and emotion regulation, given the LPP’s role in higher-order attentional processes toward affective stimuli^60,61^ and emotion regulation strategies^68–71^.

In sum, the ERPs N170, P300, and LPP frequently show atypical patterns in ASD, potentially underlying its symptomatology, including deficits in empathy and emotion regulation. The observed alterations in EEG activity suggest a potential avenue for intervention, with EEG neurofeedback presenting a promising approach to address these abnormalities, as research has shown its ability to normalize EEG rhythms in ASD^77^. Neurofeedback has gained increasing attention as a non-invasive method for modulating brain activity. By providing individuals with real-time feedback on neural signals, typically recorded via EEG or fMRI, neurofeedback enables the self-regulation of specific brain patterns, which can lead to improvements in attention^78^, emotion regulation^79^, and mental health^80^. Recent advances in this field emphasize the importance of understanding individual differences in neurofeedback responsiveness, as well as the mechanisms through which neurofeedback induces neural and behavioral change. For instance, alpha neurofeedback has been shown to enhance spatial attention by enabling participants to actively modulate alpha-band activity, resulting in persistent gains in cognitive performance^78^. Moreover, neurofeedback has demonstrated the capacity to improve selective attention in complex environments by reinforcing feature-specific neural processing^81^. Together, these findings underscore neurofeedback’s potential as a flexible and adaptive tool for both investigating and enhancing human cognitive and emotional functioning. While Holtmann et al.^82^ found no clear evidence supporting neurofeedback for ASD, suggesting that any observed benefits may be due to reductions in symptoms related to Attention deficit hyperactivity disorder rather than direct improvements in ASD core features, more recent reviews^83–85^, drawing on an expanding body of ASD research, have generally concluded that neurofeedback shows potential for alleviating ASD symptoms, underscoring the need for further investigation.

Most of the studies reviewed focused on theta/beta or mu rhythm protocols. In contrast, slow cortical potential (SCP) neurofeedback training remains rather underexplored in the ASD literature. Since SCP neurofeedback training has been shown to increase contingent negative variation^86^, and thus enhancing anticipatory attention, it may help normalize N170 activity and improve early visual face processing. Additionally, as SCP training has been found to enhance ACC activity^87^a region that exhibits reduced activation during social tasks in ASD^33^, it may help strengthen top-down attentional control. Strengthening this mechanism may enhance regulating attention, integrating social cues, and facilitating effective facial processing. Building on this, and assuming that reduced N170 amplitudes observed in ASD may reflect deficits in empathic competencies, we anticipate a more pronounced (i.e., more negative) N170 amplitude following SCP neurofeedback training compared to pre-training levels, indicating improvements in the early stages of face processing.

By enhancing attentional regulation, SCP training may decrease the need for additional attentional allocation, leading to a more efficient and reduced P300 response. Since elevated P300 amplitudes have been directly linked to ASD symptom severity^66^, we expect a reduction in P300 amplitude after SCP neurofeedback training. Furthermore, by enhancing anticipatory attention and ACC activity it could promote more efficient neural processing of affective stimuli, minimizing the prolonged attentional engagement typically reflected in LPP amplitudes. As Keifer and colleagues^66^ also reported that enlarged LPPs are associated with ASD symptomatology, we predict a reduction in LPP amplitudes following training, reflecting more efficient neural responses to affective stimuli.

Given the potential for non-specific effects in neurofeedback^87^, the use of objective measures is essential for accurately evaluating its effectiveness. Since neurofeedback is grounded in biological mechanisms, assessing physiological outcomes is crucial for directly examining the neural processes influenced by the treatment. Affective and cognitive empathic processes are often measured subjectively using tools like the Interpersonal Reactivity Index and more objectively through behavioral assessments such as the Multifaceted Empathy Test. However, to date, no existing task has integrated the objective measurement of empathy-related ERPs with behavioral assessments of different empathy facets.

## Methods and material

### Experimental design

This study is based on a randomized, controlled pre-post-test trial, investigating changes in the core symptomatology of ASD after an EEG based neurofeedback training (including the analysis of the neural dynamics of the training process) compared to treatment as usu﻿al^88,89^.

The current study extends the basic project by exploring possible changes in empathic components on the electrophysiological level before and after the neurofeedback training. For that purpose, behavioral data and event related potentials (namely the N170, P300 and the LPP) are analyzed during the execution of the Multifaceted Empathy Test for adolescents (MET-J^90^ before and after each of the two interventions (the experimental neurofeedback intervention versus the control -treatment as usual- intervention) in autistic adolescents.

### Participants

The total sample consisted of 41 male autistic adolescents, recruited from the Department of Child and Adolescent Psychiatry at the Medical University of Vienna (Austria) and the Austrian Autism Association (Vienna, Austria). Inclusion criteria required a diagnosis of ASD by an experienced clinician using either the Autism Diagnostic Observation Schedule^91^ or/and the Autism Diagnostic Interview-Revised^92^, alongside an IQ score above 70, measured with Wechsler Intelligence Scale for Children^93^. Only right-handed, male participants aged 12.00 to 17.11 years, with a proficient command of German were included. Exclusion criteria included a history of severe brain injury, comorbidities such as neurological, psychotic, or obsessive-compulsive disorders, simultaneous participation in other studies or previous neurofeedback experience. Medication status, dosage, and other therapy plans were monitored and recorded, with families asked to maintain constant treatment levels during the study to avoid influencing SCP neurofeedback training outcomes.

**Fig. 1:**
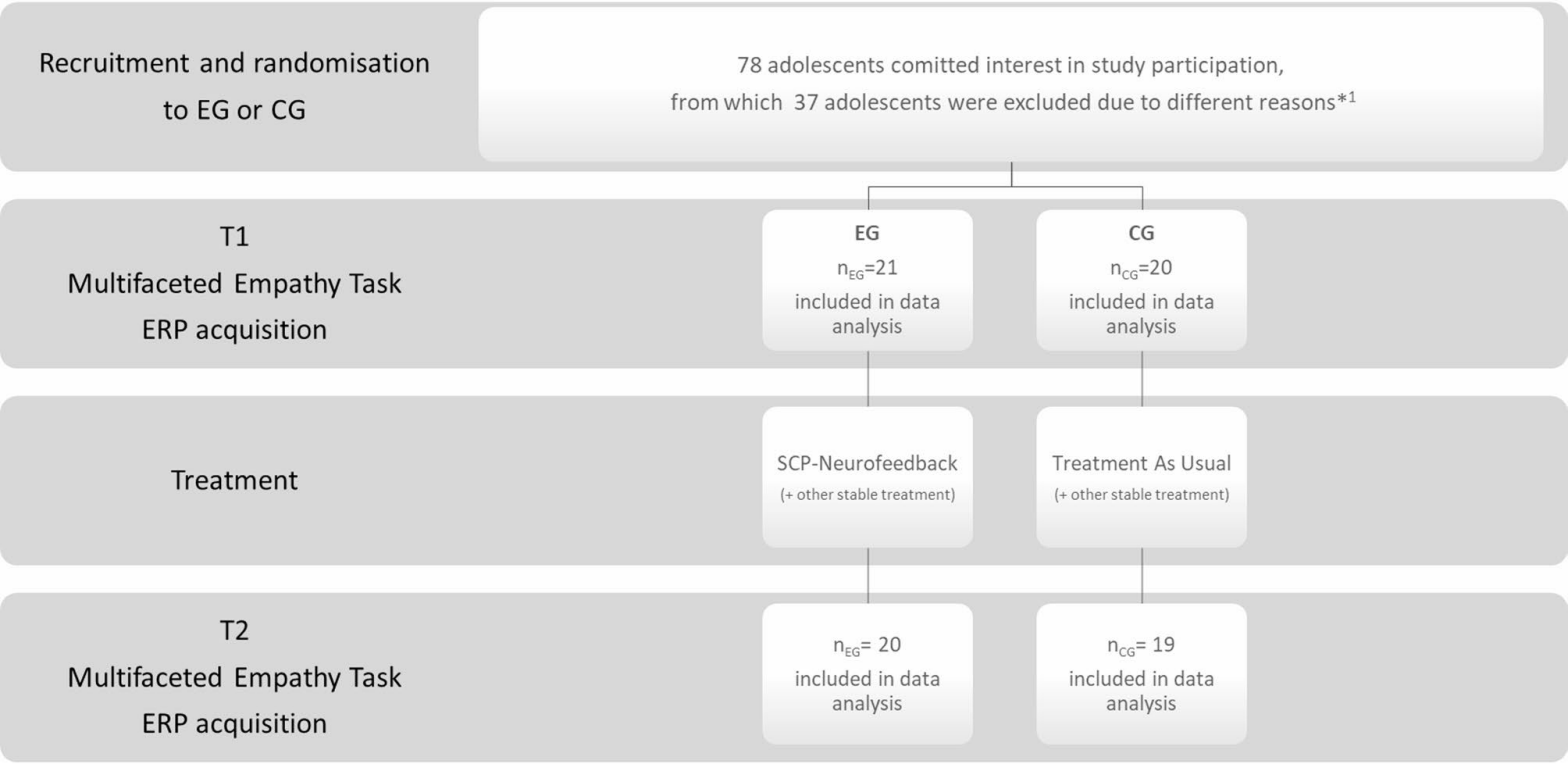
Study Design and Procedure. After randomization no drop out or losses occurred. For two participants (one of each group) Event Related Potentials (ERP) data at T2 were not recorded due to technical reasons. T1 = ERP measures before treatment, T2 = ERP measures after treatment. Group: CG = Control Group; EG = Experimental Group. In case of any other treatment already in progress, participants of both groups (EG and CG) were allowed to continue, if the usual treatment remained stable. *^1^Different reasons for exclusion were: Lost of study motivation (*n* = 12), IQ below 70 or Insufficient knowledge of German (*n* = 8), other neurological / medical conditions (*n* = 7), left-handed (*n* = 5), not aged between 12,00–17,00 years (*n* = 2), relocation or change of school (*n* = 2), former neurofeedback training (*n* = 1).

For the initial project (see^94^, which focused on changes in the core autistic symptomatology and the neural dynamics of the training process, the sample size was determined based on a power analysis for the related ANOVA (alpha = 0.05, repeated measures, within-between interactions), with an estimated effect size of 0.25. Given ASD’s heterogeneous nature, intervention effect sizes are often smaller compared to neurotypical populations. Therefore, a 0.25 effect size represents a conservative yet clinically meaningful threshold for detecting changes in this population. The power analysis revealed an actual power of 0.869 for a total sample size of 40 study participants, which was considered as sufficient. This calculation ensured that the study would be sufficiently powered to detect within-between interactions. Furthermore, the neurofeedback intervention included 24 sessions, yielding 2,880 trials per participant and a cumulative total of 115,200 trials. This robust dataset is deemed adequate for providing reliable results. For more details on the sample composition, refer to^88,89^ and Fig. 1.

### Procedure and randomization

Starting with an initial screening and clinical diagnosis of ASD, all inclusion and exclusion criteria were controlled for. Upon successful study inclusion, a structured procedure of a pairwise randomization followed: first, those two participants who applied to take part in the study in succession were defined as a pair (sequential pairing). For each pair, the names of two participants were written on separate papers and placed into individual envelopes, which were then deposited in a box. An experimenter or a designated staff member then blindly drew one envelope from the box. According to the predetermined allocation rule, the first-drawn name was assigned to the experimental group, ensuring that no subjective influence affected the process (sequence generation); the other participant was assigned to the control group. This process was repeated as many times as necessary, until the intended group size was achieved.

After pre-measures, one of two intervention started: either an experimental neurofeedback group (EG_NF_: *n* = 21) or a treatment as usual (TAU) control group (CG_TAU_: *n* = 20).

Participants in the experimental group underwent 24 neurofeedback training sessions based on SCP each lasting about 60 min, twice a week for three months. The training was divided into two phases, each consisting of 12 sessions, with a seven-day break in between. The control group received treatment as usual (TAU), which included four counseling sessions during the same period, where current problems, strengths, weaknesses, and other relevant topics were discussed without following any specific therapeutic plan. All participants of both groups (EG and CG) were allowed to continue any other usual care or existing therapy (including psychopharmacological and psychotherapeutic treatments), if it was kept stable. After 13 weeks, post-measurements were conducted in the same order as the pre-measurements. Throughout all phases of the study, sugar-free and tooth-friendly treats were given to all participants regardless of their individual performance. The quantity of these rewards was standardized, and at the end of the study, both CG and EG received the same amount. Upon completing the entire examination, all participants received an internal certificate from the ABC BRAIN LAB and a final surprise. All pre- and post-intervention measurements, as well as SCP neurofeedback training sessions, were conducted at the ABC BRAIN LAB at the Department of Child and Adolescent Psychiatry, Medical University of Vienna (Austria), between May 1, 2017, to March 12, 2020. The study received approval from the ethics committee of the Medical University of Vienna in accordance with the Declaration of Helsinki and was pre-registered on 20/04/2017 in Deutsches Register Klinischer Studien (DRKS-IDDRKS00012339). We adhered to the CONSORT reporting guidelines^75^.

### Neurofeedback training

Neurofeedback training was conducted using a 22-channel Theraprax Q-EEG system (NeuroConn GmbH, Ilmenau, Germany) equipped with seven active electrodes based on the 10–20 EEG system (FCz, REF, GND, VEOG I, VEOG II, HEOG I, and HEOG II). Additionally, pulse, respiration, temperature, and galvanic skin response were monitored during each session. Each neurofeedback session began with an eye calibration task at VEOG I, VEOG II, HEOG I, and HEOG II, which facilitated online eye movement artifact correction. SCPs were recorded at FCz and feedback was provided to participants using graphical objects displayed on a monitor. Each session comprised three training blocks with a total of 120 trials. Each trial began with a triangle pointing upwards for a required negative SCP shift and downwards for a required positive SCP shift. Participants were instructed to move the object in the desired direction using their individual strategies. Successful cortical activity changes were rewarded with a sun symbol displayed on the screen and verbal reinforcement from the instructor. The SCP neurofeedback training design is described in detail in previous publications^88,89^ as well as in the Supplementary Material in Sect. 1.1.

### Pre-post measure: the multifaceted empathy test for adolescents (MET-J)

Before and after experimental intervention (SCP neurofeedback training / Treatment as usual), the MET-J was conducted with all adolescents.

**Fig. 2:**
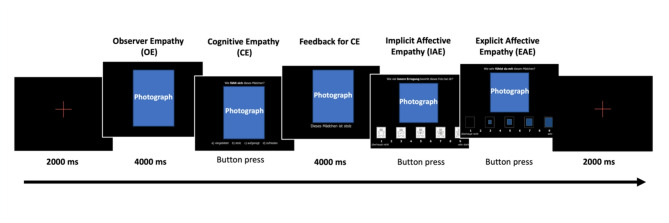
Schematic depiction of one trial in the adapted version of the MET-J. The question for CE was: “What does the person feel?”; The question for IAE was: “How much inner excitement does this photo cause in you?”; The question for EAE was: “How much do you empathize with this person?”. The blue squares are place holders for the emotional photographs.

The MET-J^90^ is a photo-based tool designed to assess the affective and cognitive aspects of empathy in adolescents. It is an adaptation of the Multifaceted Empathy Test originally developed for adults by Dziobek and colleagues^7^. This test was modified by incorporating photos and vocabulary suitable for adolescents, targeting individuals aged 12 to 17 years. In this study, an extension and adaptation of the MET-J was used to provide electrophysiological measures during the MET-J in addition. Details on the adaptation of the MET-J can be found in the Supplementary Material in Sect. 1.2. In each trial participants are first asked to observe the photograph (observer empathy, OE). In the subsequent step, they are required to infer the mental state of the individual depicted in the image (cognitive empathy, CE) and indicate which emotion is presented. The depicted emotions include sadness, anger, fear, happiness, cheerfulness, and variations of these emotions with different intensities. After participants provide their answers, they receive feedback on the accuracy to ensure that the subsequent questions are based on a consistent mental state across subjects. Additionally, participants are asked to rate their level of arousal for each image using the Self-Assessment Manikin, a nine-point visual analogue scale that ranges from “very calm” to “very aroused” (implicit affective empathy, IAE). In the final step, participants also rate the degree of empathetic concern they feel for the person in the image, using a nine-point Likert scale ranging from “not at all” to “very strong” (explicit affective empathy, EAE). Figure 2 provides an example of the adapted MET-J, showing the conditions and associated time frames.

### EEG recording and preprocessing

EEG was recorded using a 22- channel Theraprax Q-EEG-System (NeuroConn GmbH, Illmenau, Germany). The electrodes were placed according to the 10–20 system at the following positions: C3, C4, Cz, F3, F7, F8, Fp1, Fp2, FCz, P3, P4, Pz, T6, O1, O2, Oz, VEOG. The left mastoid was used as reference and the right mastoid as ground. Electrooculography was measured by placing electrodes above and below the left eye for blinks and vertical eye movements and at the outer canthi for horizontal eye movements. Electrode impedances were kept below 3 kOhm and the sampling rate was 128 Hz. All EEG data were further processed using Brain Vision Analyzer 2 (BrainProducts GmbH, Gilching, Germany). First, a 50 Hz notch filter and a low-pass filter of 0.1 Hz was applied to the signal. Then, another high-pass filter of 30 Hz and an additional low-pass filter of 1 Hz was added. Ocular correction was preformed using Independent Component Analysis based on the Infomax restricted algorithm. The ocular artefacts were additionally corrected offline based on^95^. The Oz electrode was discarded from analysis due to low data quality. Details on the data quality check are presented in the Supplementary Material in Sect. 1.3.

### ERP data extraction and analysis

For ERP extraction, the data were first segmented based on the timing of MET-J. For this analysis, OE was the segment of interest with a total duration of 4000 ms as only the emotional photograph was presented and no motor response was required. These segments of interest were baseline corrected and the signal was averaged across all 14 trials. N170 was averaged across Fz, Cz, Pz^96^; P300 was averaged across P3, P4, Pz^66^; LPP was averaged across O1, O2^66^. Local maximum (peak) and latency, and area under the curve (AUC) were calculated for all three ERP components. Time epochs were defined based on previous literature. N170 was analyzed within the 120–400 ms window^56^, and P300 within 200–600 ms^66,97,98^. LPP was divided into early (300–600 ms), middle (600–1000 ms), and late (1000–2000 ms) phases, following the definitions by Dennis & Hajcak^73^.

### Statistical analysis

For the analysis of the treatment effect on ERPs (peaks, AUC, and latencies), two-way ANOVAs were calculated with the ERP variable as the dependent variable and group (experimental vs. control group) and time (pre vs. posttreatment) as fixed factors. Here, three participants in total were removed from further analysis due to low data quality.

For the behavioral data analysis, the stimuli were separated based on their emotional valence, negative emotions (negEmotions) and positive emotions (posEmotions). Reaction times and accuracy rates during CE were extracted as well as the sum scores of ratings in IAE and EAE.

For the treatment effect on behavioral parameters two-way ANOVAs were calculated as well. In a subsequent step, we have conducted Pearson product-moment correlation to assess the relationship between change scores in ERPs (pretreatment minus post treatment) with change scores in behavioral parameters (pretreatment minus posttreatment). For two participants (one of each group) behavioral data during post treatment measurements were not recorded due to technical reasons.

All statistical analysis were performed in R version 4.3.3.

## Results

### ERP (peak amplitude, area under the curve, latency)

**Fig. 3:**
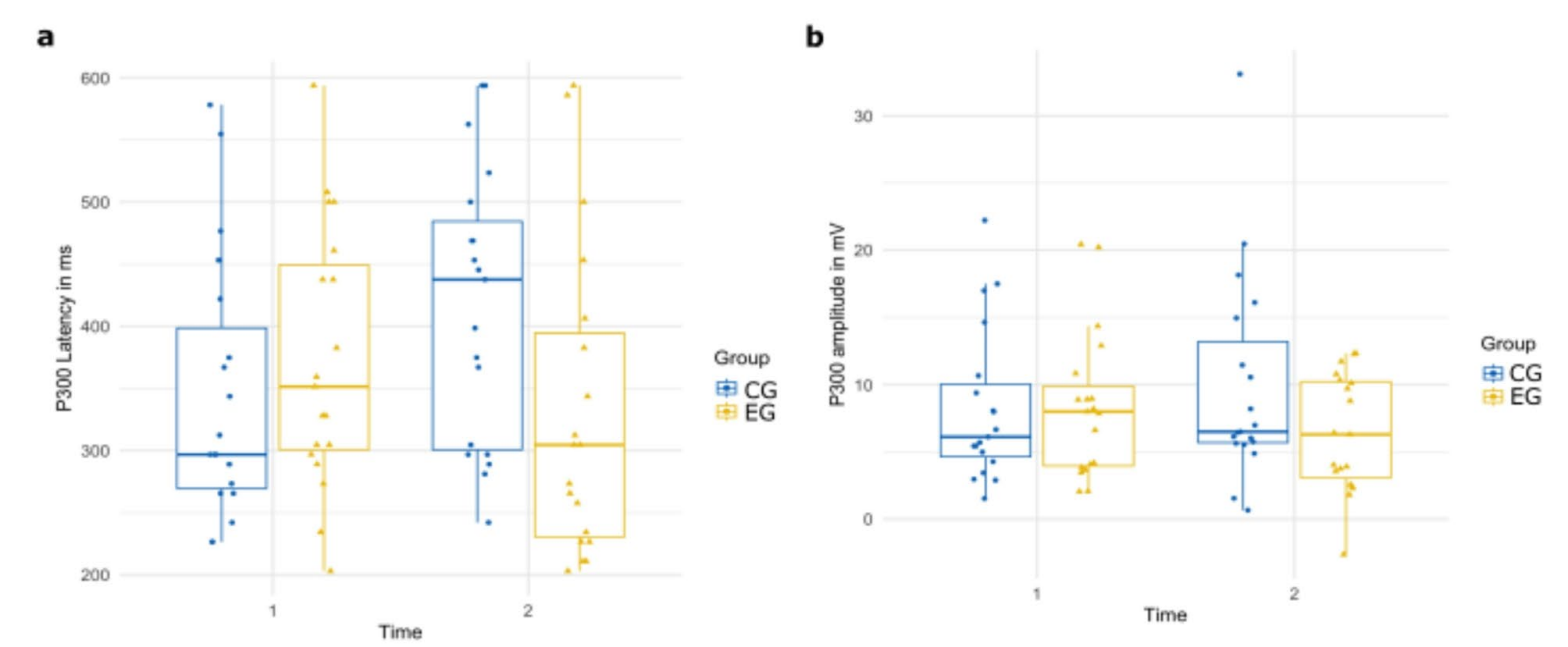
Boxplots depicting the distribution for (**a**) P300 latency and (**b**) P300 peak amplitude. Time: ”pre”= ERP measures before treatment (SCP Neurofeedack Training and TAU), “post”= ERP measures after treatment. Group: CG = Control Group; EG = Experimental Group.

**Fig. 4:**
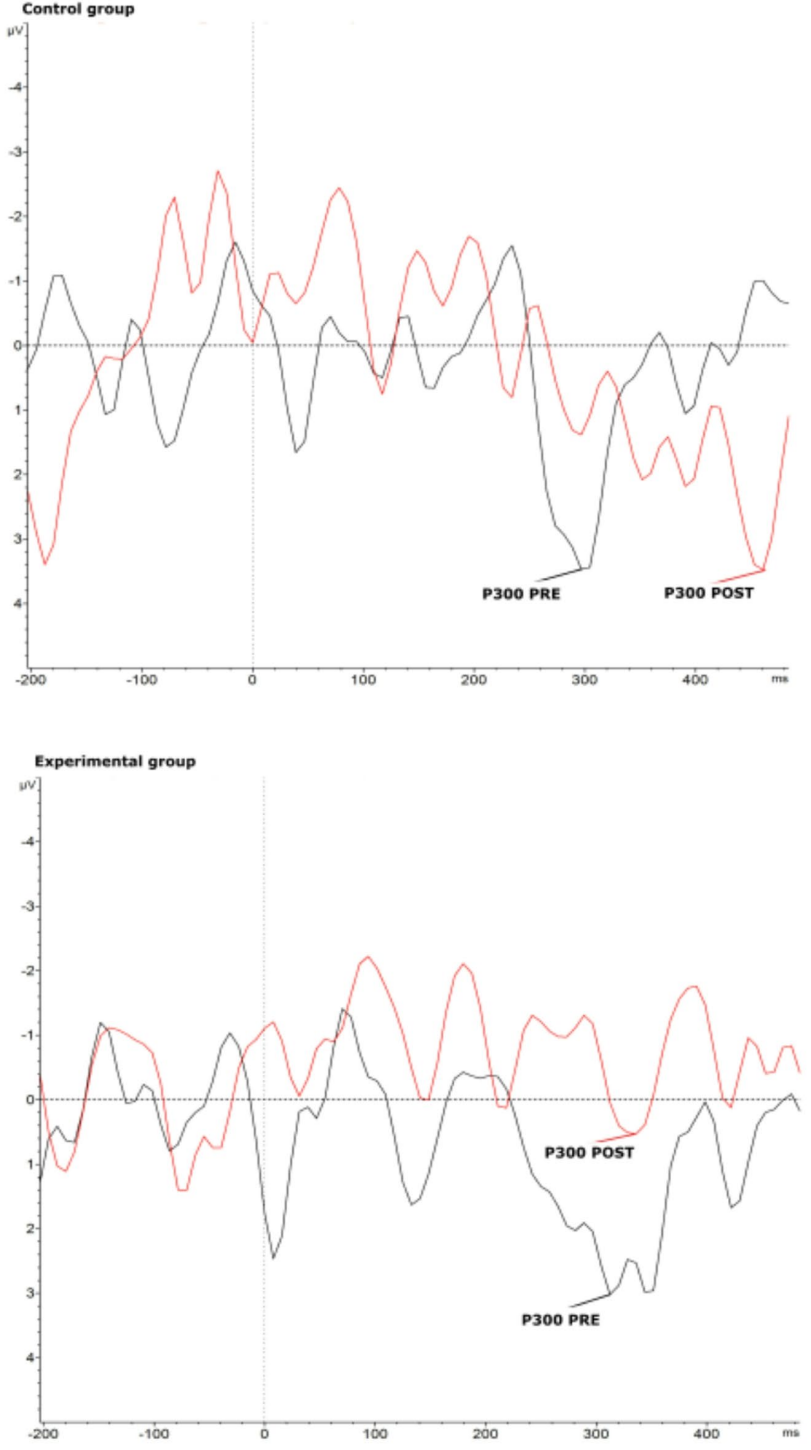
Grand Average P300 waves from and control and experimental group; black = pre treatment (SCP neurofeedback training or TAU), red = post treatment.

The results of the two-way ANOVA revealed a significant interaction between time and group on P300 latency, *F*(1, 72) = 4.79, *p* = .03, *η²*_*p*_= .06). Univariate normality of the data was given as checked by the QQ-Plot, as well as homogeneity of variances (Levene’s test: *p* = .92). Partial eta squared (*η²*_*p*_) indicates moderate effect size. Post-hoc tests indicated that participants in the control group exhibited longer P300 latencies for emotional pictures following TAU (post: *M* = 415.71 ms, SD = 110.84, 95% CI [365, 467]), compared to their baseline (pre: *M* = 345.39 ms, *SD* = 105.96, 95% CI [294, 397]). In contrast, the experimental group demonstrated a reduction in P300 latencies for emotional pictures following SCP neurofeedback training (post: *M* = 331.41 ms, *SD* = 124.06, 95% CI [280, 383]), compared to their baseline (pre: *M* = 373.36 ms, *SD* = 105.45, 95% CI [322, 424]). Post hoc tests did not reach significance level with correction for multiple testing. The two-way ANOVA for peak amplitude and AUC for P300 did not reveal significant interactions between time and group. The post-hoc analysis for P300 peak amplitude showed a trend as P300 peak amplitude was reduced after SCP neurofeedback training in the experimental group (pre: *M* = 8.39 µV, *SD* = 5.45, 95% CI [5.68, 11.11]; post: *M* = 6.34 µV, *SD* = 4.35, 95% CI [3.62, 9.06]), whereas in the control group P300 peak amplitude was increased after TAU (pre: *M* = 8.26 µV, *SD* = 5.70, 95%, CI [5.54, 10.98]; post: *M* = 9.95 µV, *SD* = 7.76, 95% CI [7.23, 12.67]). These post hoc analysis are depicted in Fig. 3. Means and standard deviations of peak amplitudes of all ERP components are presented in Table 1, while the resulting ERP plot of P300 is depicted in Fig. 4.

For the remaining peak amplitudes and latencies, as well as for AUC and latency, no significant results were obtained.

**Table 1:** Descriptive statistics for peak amplitudes.

ERP	Time	*n*	*n*	Mean (SD) µV	Mean (SD) µV
		CG	EG	CG	EG
P300	*pre*	19	19	8.26 *(5.70)*	8.39 *(5.45)*
	*post*	19	19	9.95 *(7.76)*	6.34 *(4.35)*
N170	*pre*	19	19	-6.07 *(5.10)*	-4.91 *(4.25)*
	*post*	19	19	-5.42 *(3.80)*	-7.25 *(5.16)*
LPP_early	*pre*	19	19	13.94 *(7.83)*	14.04 *(7.36)*
	*post*	19	19	13.09 *(5.01)*	13.41 *(7.23)*
LPP_middle	*pre*	19	19	9.55 *(6.54)*	9.34 *(5.68)*
	*post*	19	19	9.76 *(4.72)*	10.09 *(6.18)*
LPP_late	*pre*	19	19	8.85 *(5.22)*	8.64 *(3.70)*
	*post*	19	19	8.32 *(4.26)*	8.26 *(4.60)*

Time: ”pre”= ERP measures before treatment (SCP Neurofeedack Training and TAU), “post”= ERP measures after treatment. Group: CG = Control Group; EG = Experimental Group. *SD* = Standard Deviation. *mV* = microvolt. *n* refers to sample size after excluding outliers. For the ERP analysis, three participants (one control, two experimental) were excluded due to low EEG data quality.

### Behavioral parameters

For the effect of treatment (SCP neurofeedback training vs. TAU) on behavioral parameters in the MET-J, the two-way ANOVA showed no significant results for reaction times and accuracy rates in negEmotions and posEmotions during cognitive empathy (CE) as well as for sum rating scores in implicit affective empathy (IAE) and explicit affective empathy (EAE) across negEmotions and posEmotions.

The Pearson product-moment correlation analysis for the assessement of potential associations between changes in ERPs (pretreatment minus post treatment) with changes in behavioral parameters showed the following significant result: There was a moderate, positive correlation between reaction time in positive emotions and peak amplitude in LPP_late, *r* = .38, *p* = .02, Fisher’s z = 0.39. When split into control and experimental groups, this effect was not present in the control group (*r* = .24, *p* = .35), but remained in the experimental group with a moderate to strong association based on the effec size (*r* = .53, *p* = .02, Fisher’s z = 0.59). Specifically, an increase in the peak amplitude of LPP_late after SCP neurofeedback training was associated with a slower reaction time in processing positive emotions., in other words a decrease in peak amplitude in LPP_late was associated with faster reaction times in processing positive emotions. The remaining change score analysis did not yield any significant results.

## Discussion

This study employed a randomized, controlled pre-test-post-test design to explore the effects of SCP neurofeedback training on emotional processing in autistic adolescents, compared to TAU. The primary outcome measures included ERPs associated with emotional processing - specifically, the P300, LPP, and N170 components - as well as behavioral assessments derived from an extended and adapted version of the MET-J^90^. Our findings revealed a significant group × time interaction in P300 latency, with the experimental group showing shorter latencies after training, whereas the control group exhibited longer latencies. However, post hoc analyses did not yield significant effects. Additionally, we observed a trend toward reduced P300 peak amplitude in the experimental group, suggesting a potential neurophysiological effect of the intervention. Beyond P300, changes in the LPP late component were associated with reaction time modifications in response to positive emotional stimuli. Specifically, an increase in late component of LPP peak amplitude following SCP neurofeedback training corresponded to slower reaction times, whereas a decrease in amplitude was linked to faster processing of positive emotions. This finding suggests a dynamic interplay between neurophysiological changes and behavioral adaptations in emotional processing.

It is important to acknowledge that this study represents an exploratory pilot investigation, and the results should be interpreted with caution. The observed effects did not survive correction for multiple comparisons, emphasizing the need for replication in larger samples to confirm the reliability of these findings. Additionally, a more detailed understanding of electrophysiological responses in adolescents is crucial for refining neurofeedback protocols and enhancing their effectiveness. Despite these limitations, our findings provide valuable preliminary insights into potential neurophysiological mechanisms underlying SCP neurofeedback training in autistic individuals. By reporting these results, we contribute to hypothesis generation and offer directions for future research, including the selection of more robust outcome measures and the optimization of training protocols for this population. Thus, while cautious interpretation is warranted, these findings add to the ongoing discussion on SCP neurofeedback training and highlight the necessity of further investigations in this area.

P300 is associated with cognitive processing, including attention, working memory, and the allocation of cognitive resources to a given task^99^. Our finding of a significant group × time interaction in P300 latency, with shorter latencies in the experimental group and longer latencies in the control group, suggests that SCP neurofeedback training may facilitate more efficient neural processing of emotional stimuli. Shorter P300 latencies have been linked to faster stimulus evaluation and decision-making, potentially reflecting improved cognitive efficiency following training^100^. However, the absence of significant post hoc effects suggests that this finding should be interpreted cautiously. Additionally, we observed a trend toward reduced P300 peak amplitude in the experimental group. While not reaching statistical significance, this trend may indicate a shift in the allocation of attentional resources. In the context of ASD, larger peak P300 amplitudes have been correlated with more severe symptoms^66^. The decreased P300 amplitude could be reflective of a more streamlined and automatic processing of emotional stimuli, where less active engagement is required due to improved neural efficiency. This would suggest that the SCP neurofeedback training facilitated a shift toward more efficient cognitive functioning, possibly contributing to improvements in emotional regulation and social cognition, both of which are commonly impaired in ASD^12^. Moreover, the decrease in P300 amplitude might indicate a reduction in hypervigilance or overprocessing of stimuli, both of which are often observed in ASD individuals^101^. Given that larger P300 amplitudes have been linked to cognitive effort, the reduced amplitude may reflect a more relaxed and adaptive cognitive state, one where excessive attention or resources are no longer necessary. However, it is important to consider alternative explanations for the observed decrease in P300 amplitude. It is possible that the training led to changes in neural circuits that modulate attention and cognitive control, independent of emotional processing. Future research should explore the specific cognitive domains affected by SCP neurofeedback training and investigate whether the decrease in P300 amplitude is associated with symptom improvement in ASD across various contexts.

The N170 is sensitive to emotional facial expressions and prior research has demonstrated that more negative N170 amplitudes are correlated with higher levels of empathy and emotional engagement^38,45^. Autistic individuals however, generally exhibit a diminished N170 amplitude, reflecting deficits in facial emotion recognition and social cognition (e.g^46^. Our hypothesis was that SCP neurofeedback training could enhance emotional processing in autistic individuals, as evidenced by a more negative N170 peak amplitude post-training. While our results showed a trend towards an increase in negativity in N170 peak amplitude after SCP neurofeedback training, the change (i.e. increase) was not statistically significant. This may be attributable to the limited statistical power of our analysis due to the remaining, small EEG data sample for final specific ERP analysis for N170, P300 and LPP. The observed trend toward a more negative N170 amplitude, albeit not significant, is still noteworthy and suggests that SCP neurofeedback training may have the potential to influence neural mechanisms related to facial emotion recognition in ASD. A more negative N170 amplitude could indicate an enhanced capacity for emotional processing, aligning with prior findings that associate this more negative deflection with increased empathy and emotional sensitivity^44^. For autistic individuals, this shift in neural activity could signify progress toward more typical patterns of facial emotion processing, which is a key area of impairment in the disorder. It is also possible that SCP neurofeedback training facilitates increased engagement with emotional stimuli by improving the regulation of underlying neural circuits involved in emotion processing.

Further, we did not observe significant changes in LPP amplitude following SCP training. One possible explanation is the design of our experimental task, which employed a passive viewing paradigm. In this task, participants were not required to actively engage with or regulate their emotional responses to the stimuli presented. Passive viewing, in contrast to tasks that explicitly demand emotional regulation or decision-making, may not evoke the level of emotional engagement necessary to elicit robust changes in LPP amplitude (e.g^69^. Without the need for participants to actively process or regulate their emotional reactions, the task may have failed to capture the full extent of potential neurofeedback-induced changes in emotional processing. Another factor that could explain the lack of significant findings is the potential mismatch between the emotional salience of the stimuli and the individual sensitivities of the participants^74^. The emotional salience of stimuli can vary greatly from person to person, and it is possible that the stimuli we used were not universally or sufficiently salient to engage the participants’ empathy to a degree that would be reflected in LPP modulation.

On the behavioral level, changes in the LPP late component were associated with reaction time changes in response to positive emotional stimuli. Specifically, an increase in the late component of LPP peak amplitude following SCP neurofeedback training corresponded to slower reaction times, whereas a decrease in amplitude was linked to faster processing of positive emotions. This suggests that individuals who exhibited greater reductions in neural responsiveness to emotional stimuli following training were able to process positive emotions more efficiently.

The utility of N170, P300, and LPP as treatment outcome indices in ASD remains uncertain, with existing evidence being both heterogeneous and limited. For example, significant reductions in N170 latency have been observed in preschool-aged autistic children following a six-week individualized response treatment, suggesting its potential as a biomarker^102^. However, results from other populations, such as children with dyslexia, have shown mixed outcomes; decreases in N170 amplitude correlated with improved reading fluency only in those with larger pre-training amplitudes^102^. These findings highlight the need for further investigation to determine the reliability and clinical relevance of these indices across different interventions and populations, including ASD.

Several limitations of our study should be considered when interpreting the results. First, the MET-J, as well as the adapted version of the MET-J used in this study, are not fully optimized paradigms for ERP measurements. The extraction of ERP components could potentially require a larger number of trials to ensure stability and reliability of neural signals. However, in clinical settings, autistic adolescents often face challenges in sustaining attention and sitting through extended experimental sessions, making it difficult to implement paradigms with a high trial count. Notably, a previous study demonstrated that the LPP component reached sufficient stability after just 8 to10 trials, suggesting that even with a limited number of trials, meaningful neural responses can still be captured^70^.

Another limitation is related to the temporal manifestation of behavioral changes induced by SCP training. While our study aimed to capture changes in emotional processing immediately after the completion of the training, it is possible that the full effects of neurofeedback, particularly those related to emotional processing and empathy, emerge later in the process. Emotional and cognitive changes often develop gradually, and their biological and behavioral expression may not be immediate. To address this, future studies should consider incorporating follow-up measurements to assess the delayed effects of SCP neurofeedback training on both neural and behavioral outcomes. Longitudinal designs with multiple post-intervention assessments would provide a clearer understanding of how these effects evolve over time and may help identify whether improvements in emotional processing manifest later than we anticipated.

To conclude, these findings underscore the potential of SCP neurofeedback training as a therapeutic intervention for modulating brain activity and improving emotional functioning in ASD. Further research is needed to confirm these findings and clarify the underlying mechanisms driving these observations.

## Supplementary Information

Below is the link to the electronic supplementary material.

Supplementary Material 1

## Data Availability

The data supporting the conclusions of this article will be made available by the authors upon request. For inquiries, please contact Lilian Konicar at lilian.konicar@meduniwien.ac.at.
