# Supplementary material for: Empathy and event related potentials before and after EEG based neurofeedback training in autistic adolescents
Source: Sci Rep. 2025 Aug 22;15:30824. doi: 10.1038/s41598-025-16767-y (PMC12370891; doi:10.1038/s41598-025-16767-y)
Supplement: Supplementary file 1 — Supplementary Material 1 [file 41598_2025_16767_MOESM1_ESM.docx]

SUPPLEMENTARY MATERIAL

Empathy and Event Related Potentials Before and After EEG Based Neurofeedback Training in Autistic Adolescents

Fietz, Julia^1,2^, Auer, Gabriel ^3,4,5^, Plener, Paul ^3,4,5,6^ Poustka, Luise ^7^ & Konicar, Lilian^3,4,5 *^

1 Max Planck Institute of Psychiatry, Munich, Germany

2 International Max Planck Research School for Translational Psychiatry, Munich, Germany

3 Department of Child and Adolescent Psychiatry, Medical University of Vienna, Vienna, Austria

4 Comprehensive Center for Pediatrics (CCP), Medical University of Vienna, Austria

5 Comprehensive Center for Clinical Neuroscience and Mental Health (C3NMH), Medical

University of Vienna, 1090 Vienna, Austria

6 Department of Child and Adolescent Psychiatry and Psychotherapy, Ulm University, Ulm, Germany

7 Department of Child and Adolescent Psychiatry and Psychotherapy, University Medical Center Göttingen, Göttingen, Germany

1. **SUPPLEMENTARY METHODS**
   1. **Neurofeedback Training**

Participants in the experimental group completed 24 slow cortical potential (SCP) training sessions, divided into two phases of 12 sessions each, with a one-week break in between. During this 7-day break, they were instructed to practice their individual training strategies at home without technical support. To facilitate this, they received a reminder card displaying their preferred training object, which was also presented on-screen during training. This approach aimed to promote the transfer and generalization of learned skills into daily life. Additionally, each participant was provided with a structured home training diary to document completed exercises, mental strategies, training contexts, and behavioral changes observed during the break.

SCP brain activity was recorded from fronto-central regions (FCz electrode) and visually represented on participants’ monitors using a graphical object. Each active regulation phase consisted of 120 trials, divided into three 8-minute training blocks with different conditions. The first and last blocks were feedback conditions, where contingent SCP activity was displayed, while the middle block was a transfer condition in which no brain activity feedback was provided.

At the beginning of each trial, a triangle appeared, indicating the required SCP shift: an upward triangle signaled a negative SCP shift (increased cortical activation), while a downward triangle indicated a positive SCP shift (inhibition of cortical activation). During the active regulation phase, participants saw a moving object (e.g., a fish or a moon) that responded to their SCP activity in real time, shifting up or down according to their brain activity. The objective was to learn to control this movement volitionally. No specific strategy was suggested; instead, participants were encouraged to develop their own approach. Successful SCP modulations were reinforced by a sun symbol at the end of each trial.

Each SCP training session lasted approximately 60 minutes, including a 20-minute preparation phase, 24 minutes of training (active regulation phase), and 16 minutes of debriefing. Preparation involved cleaning the skin with an antiseptic spray, applying an abrasive gel for optimal signal quality, and attaching silver/silver chloride electrodes with conductive paste. Once connected to the amplifier, the training phase commenced. Each session began with an eye movement calibration task to enable real-time artifact correction and minimize eye movement influences.

Each SCP training session consisted of 120 trials, distributed across three training blocks (40 trials per block, 8 minutes each). The first and last blocks provided feedback (displaying SCP activity), whereas the middle block served as a transfer block without visual feedback. This design was intended to promote skill transfer from the training context to real-life situations, aligning with the principles of operant conditioning.

Each trial comprised a 2-second baseline followed by an 8-second active regulation phase. Throughout the training, peripheral physiological measures, including skin conductance, heart rate, respiration, and temperature, were recorded.

Following established neurofeedback protocols for attention-deficit/hyperactivity disorder (Strehl et al., 2017), the initial training phase focused on general volitional modulation of brain states with an equal distribution of required negativity (50%) and positivity (50%). After the training break, the protocol shifted toward disorder-specific regulation, with 80% of trials requiring negativity and 20% requiring positivity. Within each block, task conditions were presented in a randomized order.

- 1. **The Adaption of the MET for Adolescents**

The total number of stimuli from 28 in the original MET were reduced to 14 stimuli in total for each person in the adapted and extended MET-J version, to maintain a feasible test duration for clinical populations, as well as to increase specificity by excluding all black-and-white photographs and stimuli with ambiguous response options. The stimuli are equally divided by positive and negative valence, sex (male and female), and age (children, adolescents, and adults).

- 1. **EEG Artefact Correction**

To ensure a systematic and objective evaluation, we utilized the "Raw Data Inspection – Automatic Inspection" function in Brain Vision Analyzer 2 (BrainProducts GmbH, Gilching, Germany). This process involved assessing multiple channels (C3, C4, Cz, F3, F4, F7, F8, FCz, Fp1, Fp2, Fz, O1, O2, P3, P4, Pz, T5, T6) based on specific criteria. The maximum allowed voltage step was set to 50 µV within a ±200 ms window around events. A minimum activity threshold of 0.5 µV was applied to defined intervals. Additionally, the maximum allowed difference of values within intervals was limited to 200 µV over an interval length of 200 ms.

**REFERENCES**

Strehl, U., Aggensteiner, P., Wachtlin, D., Brandeis, D., Albrecht, B., Arana, M., ... & Holtmann, M. (2017). Neurofeedback of slow cortical potentials in children with attention-deficit/hyperactivity disorder: a multicenter randomized trial controlling for unspecific effects. *Frontiers in Human Neuroscience*, *11*, 135.
